# Encouraging Clinical Evolution of a Pediatric Patient With Relapsed Diffuse Midline Glioma Who Underwent WT1-Targeting Immunotherapy: A Case Report and Literature Review

**DOI:** 10.3389/fonc.2020.01188

**Published:** 2020-07-24

**Authors:** Yoshiko Hashii, Yoshihiro Oka, Naoki Kagawa, Naoya Hashimoto, Hiroyuki Saitou, Syogo Fukuya, Mizuki Kanegae, Sayaka Ikejima, Yusuke Oji, Keiichi Ozono, Akihiro Tsuboi, Haruo Sugiyama

**Affiliations:** ^1^Department of Cancer Immunotherapy, Osaka University Graduate School of Medicine, Suita, Japan; ^2^Department of Cancer Stem Cell Biology, Osaka University Graduate School of Medicine, Suita, Japan; ^3^Department of Respiratory Medicine and Clinical Immunology, Osaka University Graduate School of Medicine, Suita, Japan; ^4^Department of Immunopathology, WPI Immunology Frontier Research Center, Osaka University, Suita, Japan; ^5^Department of Neurosurgery, Osaka University Graduate School of Medicine, Suita, Japan; ^6^Department of Neurosurgery, Graduate School of Medical Science, Kyoto Prefectural University of Medicine, Kyoto, Japan; ^7^Department of Pediatrics, Osaka University Graduate School of Medicine, Suita, Japan; ^8^Department of Functional Diagnostic Sciences, Osaka University Graduate School of Medicine, Suita, Japan; ^9^Department of Cancer Immunology, Osaka University Graduate School of Medicine, Suita, Japan

**Keywords:** WT1 peptide vaccine, immunotherapy, diffuse midline glioma, relapse, WT1-specific cytotoxic T cells, tetramer assay, ELISpot assay, delayed-type hypersensitivity

## Abstract

Diffuse midline glioma (DMG) in children is a highly aggressive, malignant brain tumor that is fatal when relapsed. Wilms tumor 1 (WT1) is a high-priority antigen target for cancer immunotherapy. We hereby report on a pediatric patient who had DMG that regrew after chemoradiotherapy and underwent WT1 peptide vaccination. A 13-year-old Japanese boy presented with vertigo, diplopia, and right hemiplegia at the initial visit to another hospital, where he was diagnosed with DMG by magnetic resonance imaging (MRI); DMG was categorized to histological grade IV glioma. The patient underwent radiotherapy and chemotherapy with temozolomide. After three cycles of chemotherapy, MRI revealed tumor regrowth that translated into deteriorated clinical manifestations. Immunohistochemically, the H3.3K27M mutation in the biopsy specimen was confirmed and the specimen was positive for WT1 protein. The patient underwent WT1-targeting immunotherapy with the WT1-specific peptide vaccine because of having HLA-A^*^24:02. Consequently, his quality of life drastically improved so much as to the extent that the patient became capable of conducting nearly normal daily activities at weeks 8 to 12 of vaccination. MRI at week 8 of vaccination revealed an obvious reduction in the signal intensity of the tumor. Furthermore, betamethasone dose could be reduced successively (4, 1, and 0.5 mg/day at weeks 4, 5, and 7, respectively) without deteriorating clinical manifestations. Best response among responses assessed according to the Response Assessment in Neuro-Oncology criteria was stable disease. Overall survival was 6.5 months after vaccination onset and was 8.3 months after relapse; the latter was markedly longer than the reported median OS of 3.2 months for pediatric patients with relapsed DMG in the literature. Modified WT1 tetramer staining revealed the WT1 peptide vaccine-induced production of WT1-specific cytotoxic T cells, and the interferon-γ (IFN-γ) ELISpot assay of peripheral blood mononuclear cells disclosed the production of IFN-γ. Delayed-type hypersensitivity test became positive. Any treatment-emergent adverse events did not occur except injection site erythema. Our pediatric patient exhibited an encouraging clinical evolution as manifested by stable disease, improved clinical manifestations, steroid dose reductions, a WT1-specific immune response, and a good safety profile. Therefore, WT1-targeting immunotherapy warrants further investigation in pediatric patients with relapsed DMG.

## Background

Diffuse midline glioma (DMG) in children is a highly aggressive, malignant brain tumor, and the median overall survival (OS) after relapse for pediatric patients with DMG is 3.2 months (1). Treatment with temozolomide and bevacizumab is effective in adult patients with malignant glioma but not in pediatric patients with relapsed DMG (2, 3). Any effective therapeutic modality for relapsed DMG has not been developed in the last few decades (4), and their prognosis remains dismal, especially in patients with the H3.3K27M mutation [overall survival (OS): 9 months] as compared with those having the H3.1K27M mutation (OS: 15 months) (5). Therefore, the development of an effective treatment for them is required.

Wilms tumor antigen 1 (WT1) was considered as a high-priority antigen target for cancer immunotherapy by the National Cancer Institute because of its high immunogenicity and oncogenicity, as well as its expression in the majority of hematologic malignancies and solid tumors (6–8). In adults with malignant brain tumors (e.g., malignant glioma), the WT1 peptide vaccine was safe and induced favorable clinical and imaging responses (9, 10). We hereby report on a pediatric patient with relapsed DMG, whose H3.3K27M mutation was demonstrated by immunohistochemistry and who exhibited an encouraging clinical evolution during WT1 peptide vaccination.

## Case Presentation

A 13-year-old Japanese boy was diagnosed with DMG by magnetic resonance imaging (MRI) at another hospital—where other primary brain stem tumors were ruled out based on MRI findings, DMG was categorized to histological grade IV glioma based on the biopsy result (H3.3K27M mutation), and the patient underwent radiotherapy (54 Gy) and chemotherapy with temozolomide (280 mg/m^2^ PO), intravenously received interferon β, and was found to have HLA-A^*^24:02 by reverse transcriptase-polymerase chain reaction. Furthermore, DMG was immunohistochemically positive for WT1 protein. The patient did not have medical or family history of particular note. After three cycles of chemotherapy at another hospital, MRI revealed tumor regrowth. At presentation to our hospital, the patient showed lightheadedness, abducens nerve palsy, the deterioration of vertigo, headache, diplopia, and right hemiplegia. Subsequently, the patient was transferred to our hospital for enrollment in a phase I/II clinical trial of WT1-targeting immunotherapy with the WT1 peptide vaccine in patients with refractory pediatric cancers (UMIN 000013252), approved by the ethics committee at Osaka University Hospital and conducted according to the Declaration of Helsinki. The steering committee of the study monitored the efficacy and safety of the regimen and assessed intervention adherence and patient tolerability. The patient received the intradermal injection of 3.0 mg of the Good Manufacturing Practice (GMP)-grade, HLA-A^*^2402-restricted, 9 mer-modified WT1 peptide vaccine (mp235–243, CYTWNQMNL; Peptide Institute, Osaka, Japan) once/twice weekly for 23 weeks. Before injection, the vaccine was emulsified with an adjuvant Montanide™ ISA 51 at a weight ratio of 1:1.

**Figure 1:**
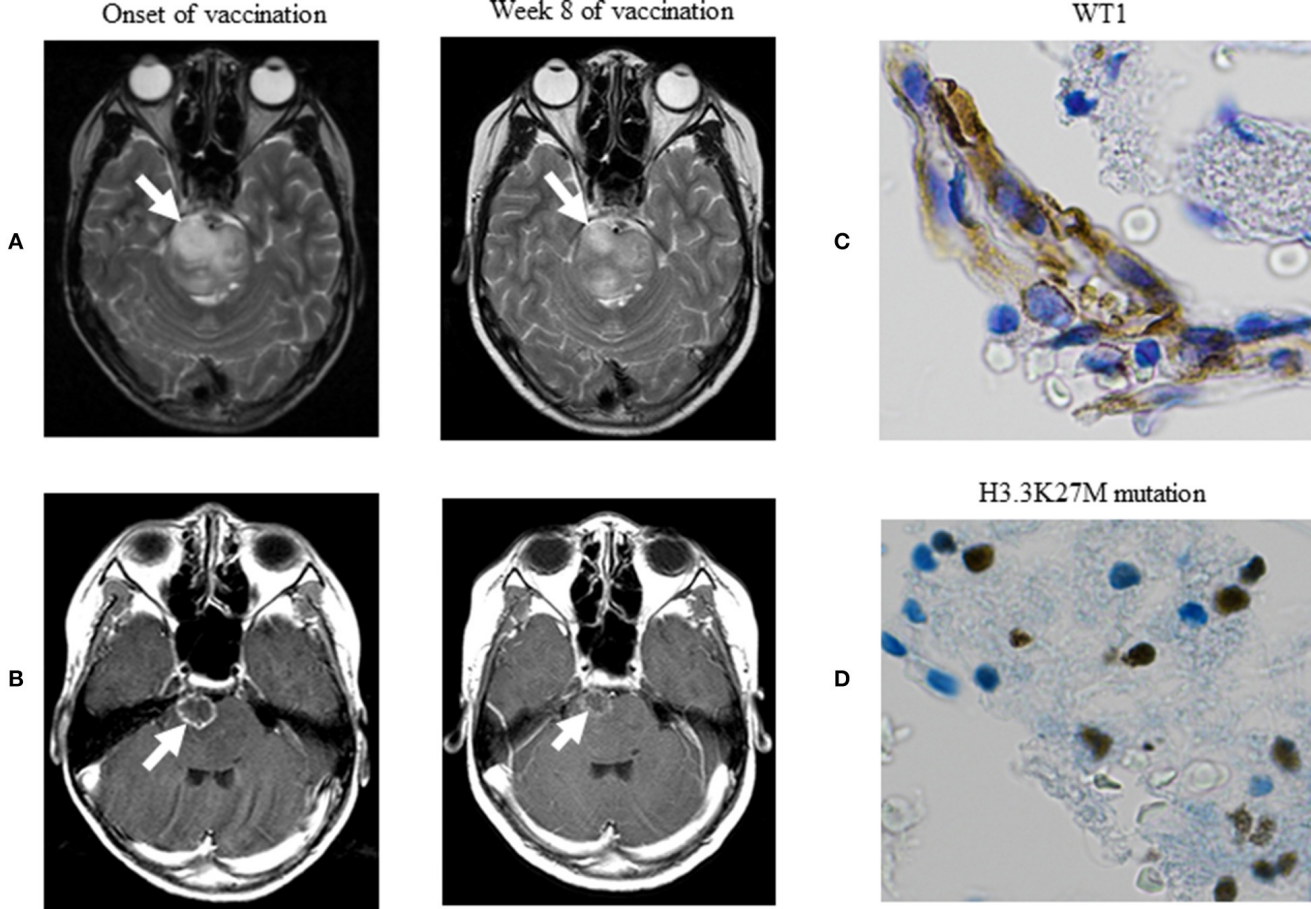
MRI of DMG. **(A)** Axial T2-weighted MR images. **(B)** Axial T1-weighted postcontrast MR images. These images demonstrate a well-circumscribed mass (arrows), and best response among responses assessed with the Response Assessment in Neuro-Oncology criteria was stable disease. **(C)** Immunohistochemical stain demonstrating WT1 protein in the nucleus and cytoplasm of tumor cells, with greater staining intensity in the cytoplasm—a previously known histopathological feature for primary astrocytic tumors (×400). **(D)** Immunohistochemical stain demonstrating the H3.3K27M mutation in the nucleus of tumor cells, displaying highly anaplastic, pleomorphic tumor cells with abnormal nuclear morphology—histopathological evidence supporting the diagnosis of histological grade IV glioma (×400). MRI, magnetic resonance imaging; DMG, diffuse midline glioma; RANO, response assessment in neuro-oncology.

At the onset of WT1 peptide vaccination, we verified tumor regrowth on a T2-weighted image. Clinical manifestations commenced to improve at week 4 of vaccination, followed by drastic improvements in his quality of life at weeks 8–12 of vaccination so much as to the extent that the patient became capable of conducting nearly normal daily activities. Along with these improvements, MRI at week 8 of vaccination revealed obvious reductions in the high signal intensity of the lesion on T2-weighted (Figure 1A) and contrast-enhanced T1-weighted postcontrast (Figure 1B) images. Betamethasone dose was reduced from 4 mg/day at the onset of vaccination to 2 mg/day at week 4, followed by reductions to 1, and 0.5 mg/day at weeks 5 and 7, respectively. Best response among responses assessed according to the Response Assessment in Neuro-Oncology criteria was stable disease (11). The patient underwent WT1-specific immunotherapy at our hospital for 23 weeks. OS was 8.3 months after relapse and was 6.5 months after vaccination onset. The patient died of progressed DMG. Any treatment-emergent adverse events did not occur except injection site erythema.

## Immunohistochemical Analysis of WT1 Protein and H3.3K27M Mutation

The paraffin-embedded sections of DMG were analyzed immunohistochemically by using the anti-WT1 protein monoclonal antibody (6F-H2 diluted 1:50, Dako) as described previously (9, 10). We newly immunohistochemically stained the preserved biopsy specimen for its presentation in the better staining condition to confirm the expression of WT1 protein and followed the staining procedures as per the manufacturer's instructions. Namely, we used the EnVision™ FLEX immunohistochemical staining system (Agilent Technologies, CA, USA) by which Dako autostainer Link 48 (Agilent Technologies) and EnVision™ FLEX Mini Kit (Agilent Technologies) were applied to automatically stain the specimen by using the primary antibody—monoclonal mouse anti-human WT1 (6F-H2) antibody (Agilent Technologies) at a dilution rate of 400 fold; a whole IgG affinity-pure antibody, AffiniPure Rabbit Anti-Mouse IgG (H+L) (Jackson ImmunoResearch, Pennsylvania, USA), was used at a dilution rate of 200 fold after the completion of the primary antibody response. Figure 1C indicates the expression of WT1 protein. In these immunohistochemical staining procedures, kidney podocytes and normal brain tissue were used to conduct control positive and negative staining, respectively.

The biopsy specimen was immunohistochemically stained to verify the occurrence of the H3.3K27M mutation as described previously (12), except Ventara autostainer BenchMark ULTRA (Ventana Medical Systems Roche, Basel-Stadt, Switzerland) and OptiView DAB IHC Detection Kit (Ventana Medical Systems Roche). Figure 1D indicates the H3.3K27M mutation in the nucleus of tumor cells.

## Immunomonitoring

### Modified WT1 Tetramer Staining of WT1-Specific Cytotoxic T Cells (CTLs), (IFN-γ) ELISpot Assay, Determination of Anti-WT1-235 IgG Antibody Titers in Serum, and Delayed-Type Hypersensitivity (DTH) Test

Cellular and/or humoral immune responses were examined by conducting the following four tests as described previously (13–16): (1) the HLA-A^*^24:02 modified WT1 tetramer-CYTWNQMNL staining of WT1-specific CTLs—WT1 tetramer^+^ CD8^+^ T cells; (2) IFN-γ ELISpot assay of peripheral blood mononuclear cells (PBMCs); (3) the determination of anti-WT1-235 IgG antibody titers in serum; and (4) DTH test with WT1 peptide.

**Figure 2:**
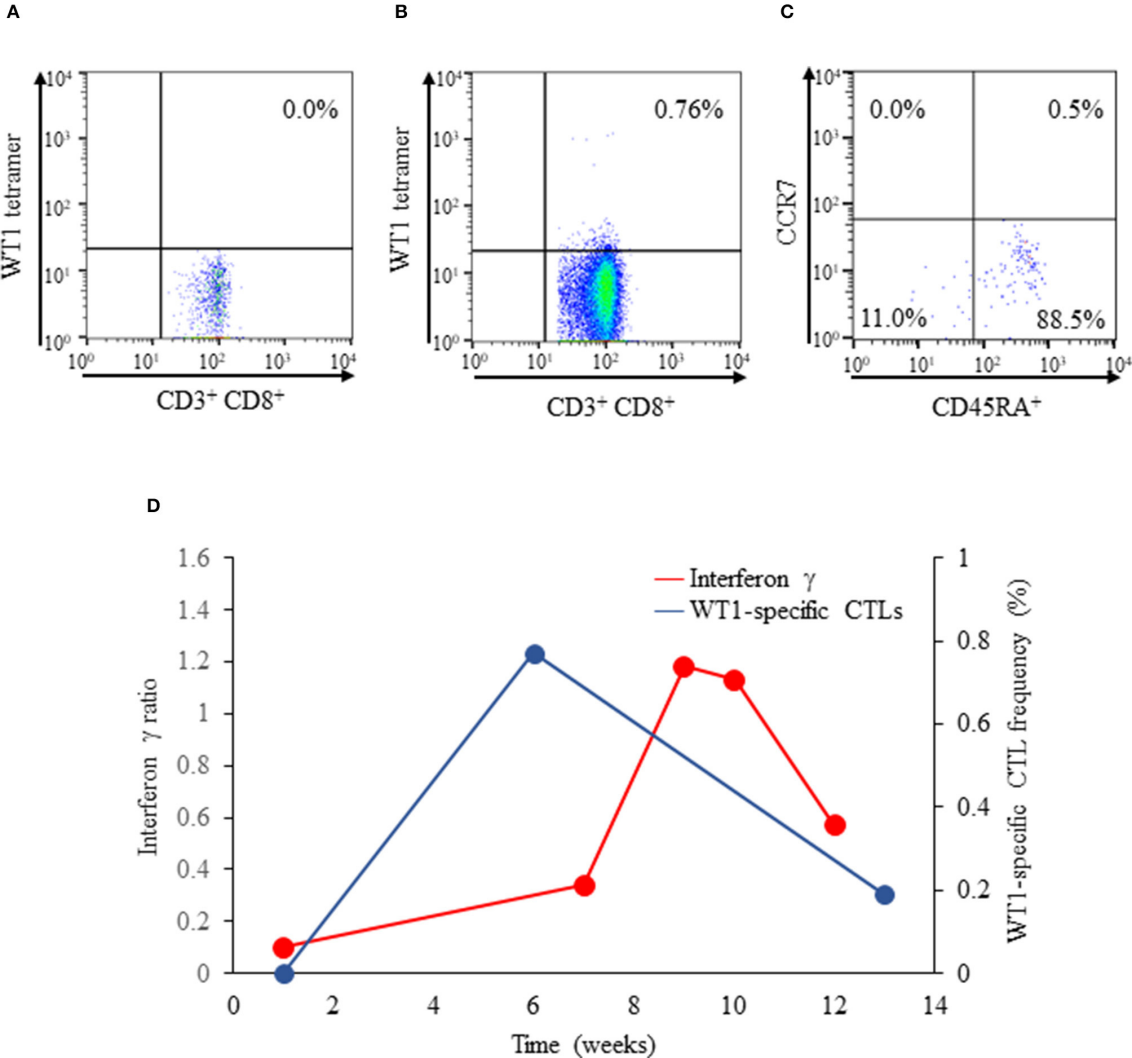
Flow cytograms of WT1-specific CTLs obtained with a flow cytometer (BD FACSCanto™ II). **(A)** WT1 tetramer^+^ CD8^+^ T cells did not emerge at the onset of vaccination (proportion of WT1 tetramer^+^ CD8^+^ T cells: 0.0%). **(B)** WT1-specific CTLs emerged at week 6 of vaccination (proportion of WT1 tetramer^+^ CD8^+^ T cells: 0.76%). **(C)** Diagram showing the phenotypes of WT1-specific CTLs: effector T cells (CD45RA^+^ CCR7^−^: 88.5%), effector memory T cells (CD45RA^−^ CCR7^−^: 11.0%), naïve T cells (CD45RA^+^ CCR7^+^: 0.5%), and central memory T cells (CCR7^+^, CD45RA^−^: 0.0%). **(D)** Interferon γ ELISpot assay of PBMCs. Isolated PBMCs were stimulated with WT1-235n (natural peptide: aa 235–243 CMTWNQMNL). The stimulated-to- unstimulated PBMC spot ratio was calculated. Interferon γ production by WT1-specific CTLs was considered positive when the ratio was >1. The frequencies (%) of WT1-specific CTLs at weeks 1 to 13 of vaccination are plotted. WT1, Wilms tumor 1; CTLs, cytotoxic T cells; PBMCs, peripheral blood mononuclear cells.

At the onset and week 6 of vaccination, WT1-specific CTL frequency was determined, and fluorescence-activated cell sorting (FACS) was conducted with a flow cytometer (BD FACSCanto™ II, BD Bioscience, San Jose, CA, USA). Data obtained were analyzed using the FACSDIVA software (BD Bioscience). Consequently, their phenotypes based on CCR7 and CD45RA expressions were determined as follows: naïve T cells (CCR7^+^, CD45RA^+^), central memory T cells (CCR7^+^, CD45RA^−^), effector memory T cells (CCR7^−^, CD45RA^−^), and effector T cells (CCR7^−^, CD45RA^+^) (17). WT1 staining of immunofuorescent T cells was conducted. Although not detected at the onset of vaccination (Figure 2A), the production of WT1-specific CTLs was induced markedly at week 6 of vaccination (0.76%; Figure 2B). Effector T cells (88.5%), effector memory T cells (11.0%), and naïve (0.5%) T cells constituted the entirety of WT1-specific T cells at week 8 of vaccination; central memory T cells (0.0%) did not emerge (Figure 2C). DTH test with WT1 peptide, which had been negative at the onset of vaccination, turned positive at weeks 8 and 10 of vaccination.

The IFN-γ ELISpot assay of PBMCs was conducted to detect IFN-γ release from CTLs. Briefly, isolated PBMCs were stimulated with WT1 235n (natural peptide: aa 235–243 CMTWNQMNL). PBMCs, with or without peptide stimulation, were smeared onto 96-well-plates precoated with the mouse antihuman IFN-γ antibody; subsequently, the smears were incubated for 18 h. The stimulated-to-unstimulated PBMC spot ratio was calculated. A ratio of >1 indicated that cytokine production from CTLs was positive. In our pediatric patient, IFN-γ production was negative at the onset of vaccination but turned positive at weeks 9 and 10 of vaccination; the positivity lasted up to week 11 of vaccination (Figure 2D), and IFN-γ production became undetectable at week 12 of vaccination. Moreover, the proportion of WT1-specific CTLs peaked at week 6 of vaccination and then gradually decreased until week 13 of vaccination (Figure 2D), presumably due to the lack of the immunologically relevant production of effector memory T cells. Anti-WT1-235 IgG antibody titers in serum were determined as described previously (15). In brief, WT1 aa 235–245 peptide was used as the capture antigen for the anti-WT1-235 IgG antibody. Anti-WT1-235 IgG antibody titers in serum were measured by enzyme-linked immunosorbent assay at the onset and at weeks 6, 8, 9, 10, 11, 12, 13, 14, and 18 of vaccination. The titers were expressed as absorbance at the wavelength of 450 nm. Consequently, anti-WT1-235 IgG antibody titers were below the detection limit at all measurement points.

## Discussion

This is the first case report on a pediatric patient with relapsed DMG, who exhibited an encouraging clinical evolution after WT1-targeting immunotherapy onset. Although best response was stable disease, MRI findings improved, clinical manifestations (e.g., decreased consciousness, vertigo, vomiting, and headache) resolved, and right hemiplegia ameliorated—all transiently at weeks 4–12 of vaccination. Furthermore, betamethasone dose was reduced successfully without deteriorating clinical manifestations. Of note was the fact that DMG remained stable owing to WT1 peptide vaccination alone despite the presence of a large residual mass of the tumor after radiochemotherapy. Namely, the WT1 peptide vaccine showed an obvious antitumor effect and extended the patient's postrelapse OS to 8.3 months as compared with the reported median of 3.2 months for pediatric patients with DMG (1).

We reviewed 14 clinical trials of immunotherapy for pediatric patients with diffuse intrinsic pontine glioma (DIPG)/DMG that were registered at ClinicalTrials.gov website in December 2019–6, 4, and 4 of which used peptide vaccines, immune checkpoint inhibitors, and other treatments, respectively (Table 1). Concretely, a peptide vaccine against the point mutation (K27M) of the *histone-3* gene (H3F3A)—a driver gene—seems to be promising and is currently under development. Rindopepimut is a vaccine that targets EGFRvIII protein; its trial in pediatric patients with DMG was discontinued due to the lack of efficacy in the phase III trial for adult glioblastoma (18). DSP7888 contains a peptide that induces WT1-specific CTLs and helper T cells; a phase I/II study of the agent is underway for the treatment of pediatric patients with DMG, grade III glioma, or grade IV glioblastoma in Japan, which used vaccines, a programmed cell death 1 receptor checkpoint inhibitor, and an immunomodulator. Four of these 14 clinical trials, which provided clinical outcomes from pediatric patients with newly diagnosed DIPG/DMG, are summarized in Table 2—(1) the glioma-associated antigen-based vaccine (2, 19) the autologous dendritic cell vaccine (3, 20) pidilizumab (21); and (4) pegylated IFN-γ-2b (22). Our pediatric patient, who had relapsed DMG, showed an encouraging clinical evolution presumably due to the following facts: (1) the vaccine is specific to tumor-overexpressed WT1 that is highly immunogenic, tumorigenic, and angiogenic, and that regulates the apoptosis of many malignant brain tumors (23)—the features that drive us to consider that the WT1 peptide vaccine suppresses tumorigenesis in the patient and tumor angiogenesis and enhances immunogenicity of the patient and tumor apoptosis; (2) steroid dose reductions were possible; and (3) the vaccine has a good safety profile. In general, greater refractoriness to treatments is observed in patients with relapsed DMG than in those with newly diagnosed DMG.

**Table 1:** Clinical trials of immunotherapy in pediatric patients with diffuse intrinsic pontine/midline glioma registered at ClinicalTrials.gov as of December 2019.

**Identifier no**.	**Status**	**Study results (reference)**	**Phase**	**Disease**	**Intervention**
NCT01400672	Completed	None available	I	Relapsed or progressive disease	Tumor lysate vaccine, imiquimod
NCT02960230	Recruiting	None available	I	Newly diagnosed	K27M peptide
NCT01058850	Terminated	None available	I	NR	Rindopepimut
NCT02750891	Recruiting	None available	I/II	Relapsed or progressive disease	DSP7888
NCT01130077	Recruiting	Available (19)	NR	Newly diagnosed	Glioma antigen peptide vaccine
NCT02840123	Active; not recruiting	Available (20)	I	Newly diagnosed	Autologous dendritic cell vaccine
NCT02359565	Recruiting	None available	I	Relapsed or progressive disease	Pembrolizumab
NCT03130959	Active	None available	Ib/II	Newly diagnosed	Nivolumab vs. ipilimumab
NCT01952769	Active; not recruiting	Available (21)	I/II	Newly diagnosed	Pidilizumab
NCT02793466	Recruiting	None available	I	Relapsed	Durvalumab
NCT00036569	Completed	Available (22)	II	NR	Pegylated interferon γ-2b
NCT03389802	Active	None available	I	Newly diagnosed, recurrent, or progressive disease	APX005M
NCT03330197	Active	None available	I	NR	Ad-RTS-human interleukin-12
NCT02502708	Recruiting	None available	I	Newly diagnosed	Indoximod in combination with temozolomide, or with radiation followed by indoximod/temozolomide combination

*NR, not reported*.

**Table 2:** Results of clinical trials of immunotherapy in pediatric patients with diffuse intrinsic pontine/midline glioma.

**Identifier no. (reference)**	**Phase**	**Disease**	**Intervention**	**No. of patients**	**Median overall survival after diagnosis**	**Adverse event**
NCT01130077 (17)	NR	Newly diagnosed	Glioma antigen peptide vaccine	26 BSG (14 BSG underwent radiotherapy and 12 BSG/HGG radiochemotherapy)	12.7 months	No grade 3 or 4 adverse events
NCT02840123 (18)	I	Newly diagnosed	Autologous dendritic cell vaccine	9 DIPG	NR^a^	Grade 3 osteomyelitis
NCT01952769 (19)	I/II	Newly diagnosed	Pidilizumab	9 DIPG	15.6 months	Grade 3 neutropenia and BP elevation
NCT00036569 (20)	II	Newly diagnosed	Pegylated interferon γ-2b	32 DIPG	351 days	Grade 3 neutropenia

*BP, blood pressure; BSG, brain stem glioma; DIPG, diffuse intrinsic pontine glioma; HGG, high-grade glioma; NR, not reported*.

a *Vaccination was completed, safety was acceptable, and a WT1-specific immune response was detected*.

A WT1-specific immune response was successfully induced as evidenced by the emergence of WT1-specific CTLs, by IFN-γ production in the IFN-γ ELISpot assay of PBMCs, and by the development of DTH after vaccination onset even during betamethasone administration. The phenotypic analysis of WT1-specific CTLs revealed that the effector T cells (88.5%), effector memory T cells (11.0%), and naïve T cells (0.5%) constituted the entirety of WT1-specific CTLs at week 6 of vaccination. The high frequency (>13%) of effector memory T cells was correlated with longer survival and higher clinical response rates in patients with advanced melanoma (24). In our pediatric patient, as high as 11.0% of WT1 tetramer^+^ CD8^+^ T cells emerged as effector memory T cells at week 6 of vaccination, and the IFN-γ ratio surpassed 1.0 at week 9 of vaccination, indicating that WT1-specifc CTLs became functional immunologically. We presume that these facts contributed, at least in part, to 8-week improvements in clinical manifestations and MRI findings.

The development of DTH to the WT1 peptide vaccine, as reported in previous studies (13, 15), indicates a good prognosis of patients with malignant brain tumors. Indeed, our pediatric patient developed DTH but was unable to produce the anti-WT1-235 IgG antibody that requires helper T lymphocytes which are involved in the immunoglobulin class switch. Therefore, co-vaccination with WT1 killer and helper peptides may be required to enhance clinical efficacy and anti-WT1 IgG antibody production.

The central nervous system (CNS), in which an immune response is considered less prone to develop owing to the blood-brain barrier, has immune privilege (25). However, a lymphatic system of the CNS, through which activated T cells can penetrate into the brain parenchyma, was recently discovered (26). In patients with glioblastoma multiforme who underwent peptide-pulsed dendritic cell-based immunotherapy that induces the considerable production of systematically activated T cells (27), the robust infiltration of CTLs and memory T cells into the intracranial tumor was associated with the prolonged survival thereof. Therefore, the increased production of systemically activated T cells and memory T cells may be important for immunotherapy to exert greater efficacy.

Our study has several limitations. First, the long-lasting efficacy of WT1-targeting immunotherapy cannot be expected for pediatric patients with relapsed DMG who have a large residual tumor mass after radiochemotherapy. Second, tumor reduction surgery, which is important for successful immunotherapy, was impossible to conduct for our pediatric patient who had unresectable DMG—the fact that was responsible, at least in part, for his poorer prognosis in comparison with adult patients with glioblastoma who underwent the surgery and for whom the WT1 peptide vaccine induced a clinical response and longer survival than the historical control at our institution (9). Third, the gene expression profiling of our pediatric patient remains to be conducted.

In conclusion, our pediatric patient who had the H3.3K27M mutation and was treated with WT1-targeting immunotherapy with the present peptide vaccine exhibited an encouraging clinical evolution, warranting further clinical research on this therapeutic modality.

## Data Availability Statement

All datasets generated for this study are included in the article.

## Ethics Statement

The studies involving human participants were reviewed and approved by Ethical Review Committee of Osaka University Faculty of Medicine. Written informed consent to participate in this study was provided by the participants' legal guardian/next of kin. Written informed consent was obtained from the individual(s), and minor(s)' legal guardian/next of kin, for the publication of any potentially identifiable images or data included in this article.

## Author Contributions

All authors conceived and designed the study, participated in writing the manuscript, and approved the final version of the manuscript. YH, NK, HSu, YOj, and KO provided study materials or patients. YH, NK, SF, HSa, and NH collected and assembled data. YH, YOk, NK, SF, and NH analyzed and interpreted data.

## Conflict of Interest

The authors declare that the research was conducted in the absence of any commercial or financial relationships that could be construed as a potential conflict of interest.
